# Three new species of *Impatiens* (Balsaminaceae) from southern Western Ghats, Kerala

**DOI:** 10.3897/phytokeys.180.66748

**Published:** 2021-08-13

**Authors:** Sindhu Arya, Murugan Govindakurup Govind, Veerankutty Suresh, Walsan Kalarikkal Vishnu, Venugopalan Nair Saradamma Anil Kumar

**Affiliations:** 1 Department of Botany, (Research centre University of Kerala) University College, Thiruvananthapuram, Kerala – 695034, India; 2 Plant Genetic Resource Division, Jawaharlal Nehru Tropical Botanic Garden and Research Centre (Research Centre, University of Kerala), Palode, Thiruvananthapuram, Kerala, India; 3 Department of Botany, Government Victoria College, Palakkad, Kerala – 678001, India; 4 Laboratory of Immunopharmacology and Experimental therapeutics, Regional Cancer Centre, Thiruvananthapuram, Kerala – 695011, India

**Keywords:** Balsaminaceae, endemic species, *
Impatiens
*, Western Ghats

## Abstract

Three new species of *Impatiens*, *Impatiensachudanandanii*, *I.danii*, and *I.shailajae*, are described from Thiruvananthapuram and Idukki districts of Kerala state (SW-India). *Impatiensachudanandanii* is similar to *I.courtallensis* and *I.herbicola*; *I.danii* to *I.goughii* and *I.shailajae* is to *I.minae* and *I.scapiflora*. The newly described taxa are readily distinguished from their allied species by unique character combinations, *viz.* shape of lateral sepal, lower sepal, dorsal petal, seed and pollen morphology. Detailed descriptions along with illustrations and photographs are provided.

## Introduction

Balsaminaceae A. Rich consists of about 1,000 species, mainly distributed in tropical Africa, Madagascar, southern India, and Sri Lanka (see e.g., Yuan et al. 2004). This family includes annual or perennial herbs (more or less succulent) or sub-shrubs. (Stevens 2012). It comprises the monotypic genus *Hydrocera* Blume ex Wight & Arn. and the genus *Impatiens* L. having variously united and characteristic petals with dehiscent fruits (Mabberley 2008; Bhaskar 2012). *Impatiens* is mainly distributed in the tropics and subtropics of the Old World, whereas few species occur in temperate regions of Eurasia and North America. Five diversity hotspots for *Impatiens* have been recognized, i.e. tropical Africa, Madagascar, southern India and Sri Lanka, the eastern Himalayas, and southeast Asia (Song et al. 2003; Yuan et al. 2004). During the past two decades, extensive contributions to the taxonomy of the genus *Impatiens* were made (Yu et al. 2015; Fischer and Rahelivololona 2015a, b, c, 2016; Fischer et al. 2017). Yu et al. (2015) divided *Impatiens* into two subgenera *Clavicarpa* and *Impatiens* with 7 sections *viz. Semeiocardium*, *Impatiens*, *Tuberosae*, *Racemosae*, *Uniflorae*, *Scorpioidae* and *Fasciculatae*. The three new species described here belong to the subgenus Impatiens characterized by 5–carpellate (rarely 4) ovary, many ovules per locule; fusiform, linear, cylindrical or clavate capsule; pollen 4–aperturate (rarely 3–aperturate), oblong, circular, elliptic or quadrate.

*Impatiens* is represented by more than 210 taxa in India, mostly distributed through the Eastern Himalayas and the Western Ghats (see e.g., Bhaskar 2012). More than 106 species are endemic to the Western Ghats, of which 80% are endangered (Bhaskar 2012). Moreover, several endemic taxa have been recently reported from various parts of the Western Ghats (Hareesh et al. 2015; Chhabra et al. 2016; Vishnu et al. 2020).

The interiors of Kerala forest ranges are bestowed with rich biodiversity and many of which warrant keen exploration. During such field explorations, in a span of two years, we came across three interesting species of the genus *Impatiens* from Thiruvananthapuram and Idukki districts of Kerala. Critical analysis of the specimens revealed that these cannot be ascribed to any known species of *Impatiens* and hence described here as new.

## Materials and methods

Extensive field surveys were conducted in Kerala during the period 2019–2021. Analysis of relevant literature (Hooker and Thomson 1860; Hooker 1875, 1904–1906, 1908a, b, 1910, 1911; Dessai and Janarthanam 2011; Bhaskar 2012; Hareesh et al. 2015; Ramasubbu et al. 2015, 2017; Bhaskar and Sringeswara 2017; Mani et al. 2018) and careful examination of preserved specimens preserved at various herbariums (TBGT, KFRI, MH, USF, K, CALI, and CMPR) (acronyms according to Thiers (2021) [continuously updated]) were undertaken to complete the study. Furthermore, distribution map was created using QGIS Version 3.14.

## Results and discussion

### Impatiens achudanandanii

Taxon classificationPlantaeEricalesBalsaminaceae

Kumar V.S.A., M.G. Govind & Sindhu Arya sp. nov.

2AB72CE5-299C-5CAE-9F3A-A3DE14CF2649

urn:lsid:ipni.org:names:77219068-1

Figs 1 , 2 , 7

#### Type.

India. Kerala, Thiruvananthapuram, Kallar forest area, along the streams of highland 8.7599°N, 77.1169°E, 1200 m a.s.l., 26 August 2019, Kumar V.S.A.., M.G. Govind & Arya.S, 1056 (holotype TBGT!, isotype MH! CALI!).

#### Diagnosis.

*Impatiensachudanandanii* is similar to *I.courtallensis* Ramasubbu, from which it differs by the color of the flowers (whitish-creamy with yellow spot at throat in *I.achudanandanii*vs. milky-white in *I.courtallensis*), the shape of the fruit, the shape, number and hairiness of the seeds (ovoid, 2–3 seeded fruit and seed glabrous in *I.achudanandanii* vs. fusiform, 3–5 seeded and with minute hairs in *I.courtallensis*), the shape of the dorsal petal (ovoid-circular in *I.achudanandanii* vs. orbicular, recurved in *I.courtallensis*), the shape of the lateral united petals (basal lobe ovate-obovate, distal lobe round in *I.achudanandanii* vs. basal lobe oblong, distal lobe spherical in *I.courtallensis*), the shape of lower sepal (saccate and tip pointed in *I.achudanandanii* vs. boat shaped and tip outwardly curved in *I.courtallensis*) and the size and color of the pollen grains (10 × 16 µm whitish-yellow in *I.achudanandanii* vs. 16 × 18 µm squarish, milky-white in *I.courtallensis*).

#### Description.

Annual, succulent, straggling, glabrous herb, 15–20 cm high; stems terete, unbranched with purple dots, nodes slightly swollen, internode elongated, 1–1.5 cm. Leaves opposite, decussate, 2–2.5 × 1–1.2 cm long, shortly petiolate, petiole 0.5 mm, coriaceous, linear, acuminate, entire, base truncate, slightly cordate, reflexed upwards, leaf margin distinctly serrate, leaf blade 1–2 mm, extra petiolar glands absent. Inflorescence 2–3 together, flowers simple, pedicellate, axillary, 2–3 mm across, whitish-creamy with yellow spot on the throat; pedicels 0.5–0.8 cm long. Sepals– lateral 2, linear-lanceolate aristate, 0.5–1 mm long, faintly nerved, white. Lower sepal boat shaped, tip of the lower sepal pointed, 1.0–1.2 × 0.5–0.8 mm, horizontal, spur minute, 0.2 mm, yellow. Petals-dorsal ovate, 1.5–1.8 × 0.5–0.8 mm, beaked, dorsally keeled, apiculate, lateral united petals stipitate, not clawed, 2 lobed, margin smooth 1–1.5 mm long, basal lobe small, ovate, distal lobe round, dorsal auricle not prominent, end sharp. Ovary ovoid, 0.5 mm long. Fruit: capsules small, ovoid, turgid, 3–5 × 2–3 mm, acute, red shaded, 2–6 seeded; seed hexagonal, smooth, compressed, 1–2 × 0.5–1 mm. Pollen grains 10 × 16 µm whitish-yellow.

**Figure 1.**
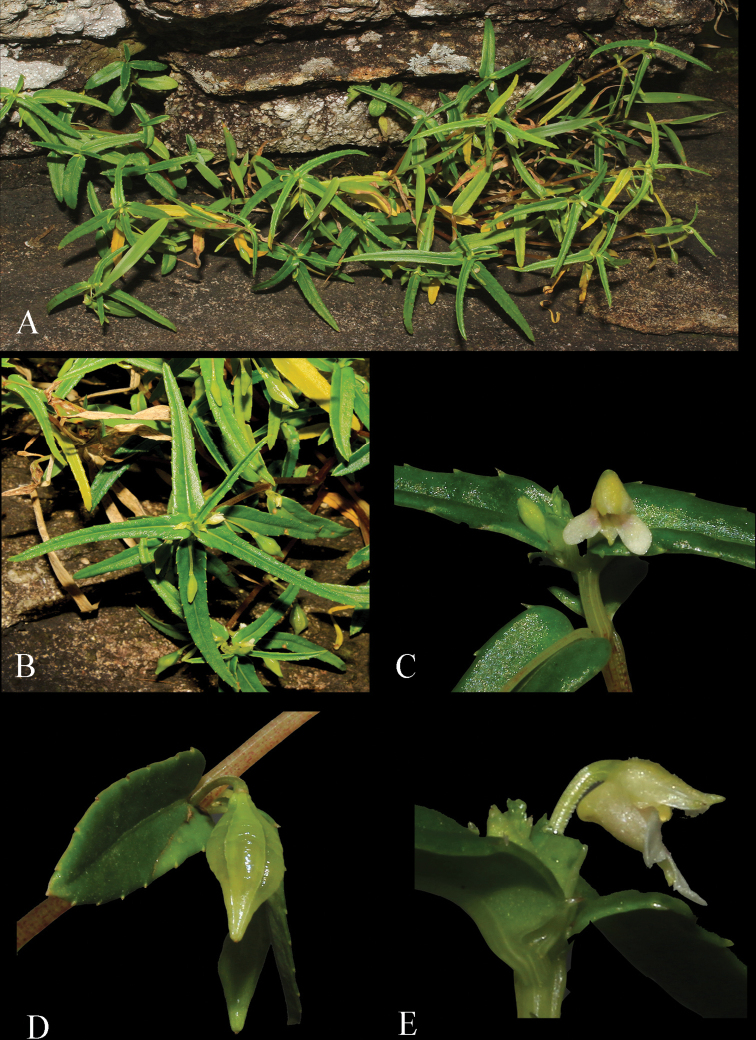
*Impatiensachudanandanii***A** habit **B** twig **C** flower **D** fruit **E** side view of flower. Photos by Govind.

#### Etymology.

*Impatiensachudanandanii* is named in honor of Mr. V.S. Achudanandan, former Chief Minister of the state of Kerala for his ardent efforts in conservation of the pristine environment of Western Ghats, especially Mathikettan shola.

**Figure 2.**
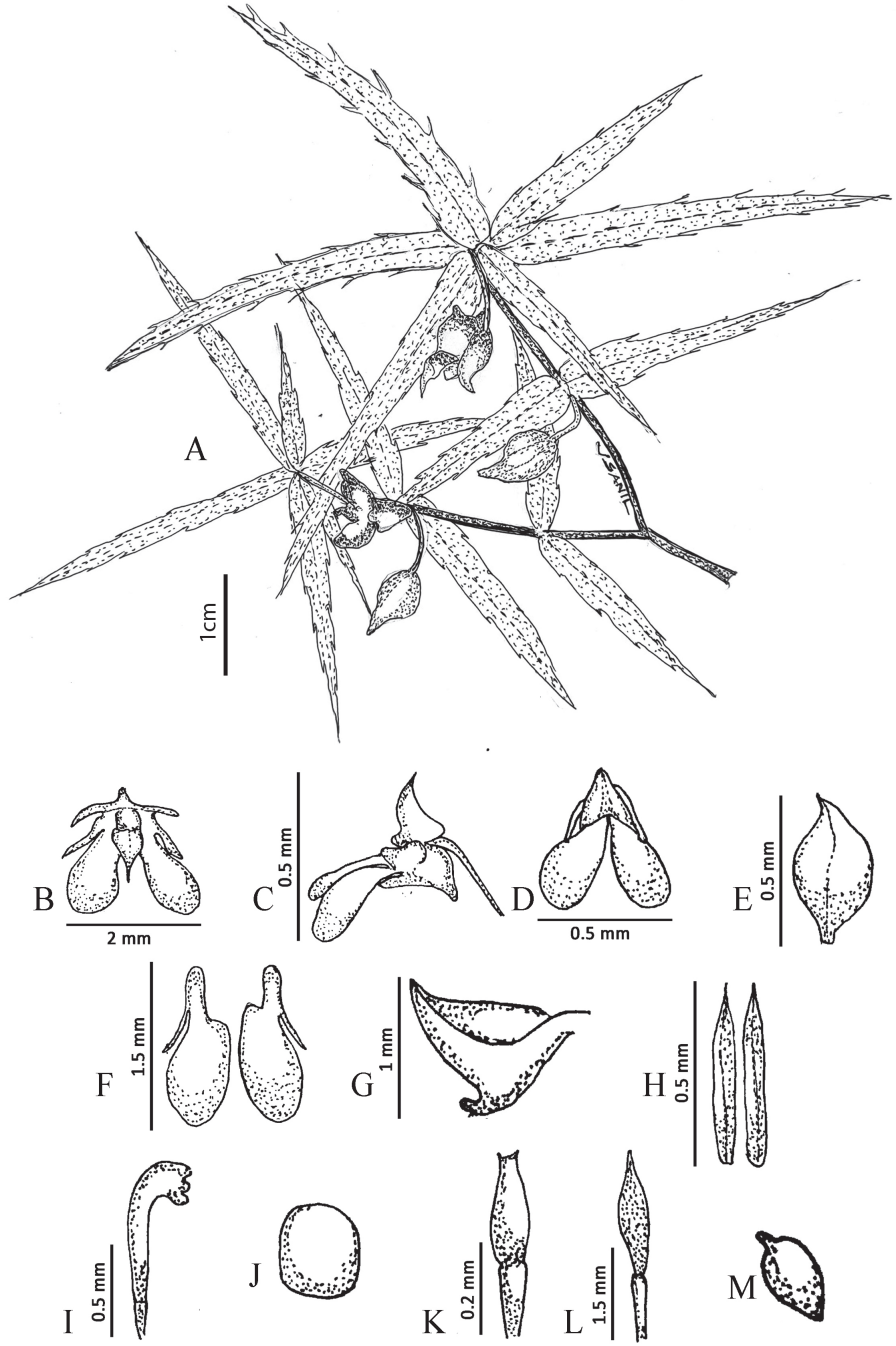
*Impatiensachudanandanii***A** habit **B** flower (front view) **C** flower (lateral view) **D** petals **E** dorsal petal **F** lateral petals **G** lower sepal **H** lateral sepal **I** stamen **J** pollen **K** gynoecium **L** fruit **M** seed. Illustrations by V.S. Anilkumar.

#### Phenology.

August to November.

#### Distribution and habitat.

*Impatiensachudanandanii* is distributed in the highlands above 1200 m. So far, the specimen has been observed only in the type locality. The populations are scattered and under the threat of grazing and other anthropogenic pressures. The plants are found to grow associated with seasonal ditches near the shade of huge rocks. Species of *Eriocaulon*, *Utriculariareticulata* and *Droseraindica* were found to grow in the nearby vicinity of this species

#### Conservation status.

*Impatiensachudanandanii* is assessed as Critically Endangered (CE) according to the IUCN categories using the criterion d (IUCN 2019). There were only three to four populations (10–15 individuals per population) observed within 1 km. The habitat of the species was severely affected by stamping of wild elephants and land-slides.

#### Other notes.

*Impatiensachudanandanii* belongs to the section Uniflorae under the subgenus Impatiens. characterized by ellipsoidal shape of seed and capsules that are short and conspicuously turgid at middle. The new species resembles *I.courtallensis*, a species reported from Courtallam hills of Tamil Nadu and also *I.herbicola*, a common high altitude species. The shape of dorsal petal, minute size of flower and the presence of spur distinguished the newly described species from its allied taxa. *Impatiensachudanandanii* is distinct from its other allied taxon *I.herbicola* with respect to spur (present in *I.achudanandanii* vs. absent in *I.herbicola*), color of the flower (whitish-creamy with yellow spot at throat in *I.achudanandanii* vs. bluish or yellowish in *I.herbicola*), size of the flower (2–3 mm in *I.achudanandanii* vs. 5–9 mm in *I.herbicola*), shape of the fruit (ovoid 2–6 seed in *I.achudanandanii* vs. gibbously ovoid with many seeded in *I.herbicola*), shape of lateral united petals (basal lobe obovate-ovate in *I.achudanandanii* vs. oblong in *I.herbicola*), shape of dorsal petal (circular, faintly keeled in *I.achudanandanii* vs. orbicular and thickly keeled in *I.herbicola*) as well as size and color of the pollen (10 × 16 µm whitish-yellow in *I.achudanandanii* vs. 21 × 23 µm, yellow in *I.herbicola*).

#### Specimen examined.

*Impatiensachudanandanii* India. Thiruvananthapuram, Kallar. 22 August 2019, Arya & Kumar V.S.A. 1057 (MH!, TBGT!); 10 September 2019. M.G. Govind 957 (TBGT!). *Impatiensherbicola* India. Thiruvananthapuram, Kallar. 22 August 2019, Kumar V.S.A. & Arya 1037 (TBGT!); 4 September 2019, Arya & Kumar V.S.A. 1097 (TBGT!).

### Impatiens danii

Taxon classificationPlantaeEricalesBalsaminaceae

M.G. Govind, Sindhu Arya, V. Suresh & Kumar V.S.A. sp. nov.

8CFAAC14-3124-58FF-9AE7-821A7C3FDED7

urn:lsid:ipni.org:names:77219072-1

Figs 3 , 4 , 7

#### Type.

India. Kerala, Idukki, Munnar, along the streams of highland 10.0889°N, 77.0595°E, 800 m a.s.l., 16 October 2019, M.G. Govind & Kumar V.S.A.., 1078 (holotype TBGT!, isotype MH! CAL!).

#### Diagnosis.

*Impatiensdanii* is similar to *Impatiensgoughii* Wt. (1831:160) but different in terms of color of flower (white with yellow blotch on the throat in *I.danii*vs. purple with white blotch on the throat in *I.goughii*), nature of peduncle and pedicel (non– sticky, smooth 3–3.5 cm in *I.danii*vs. sticky, viscous 7–8 cm in *I.goughii*), bracts (absent in *I.danii* vs. present and minute in *I.goughii*), shape of spur (curved and equal or longer than flower in *I.danii* vs. straight and shorter than flower in *I.goughii*), shape of dorsal auricle (short, lanceolate and equals the length of wings in *I.danii*vs. long and filiform and half the length of wings in *I.goughii*), capsule (ovate in *I.danii*vs. ellipsoidal in *I.goughii*) and shape of seed (ellipsoidal with bands of hairs in *I.danii*vs. ovoid with short hairs in *I.goughii*).

**Figure 3.**
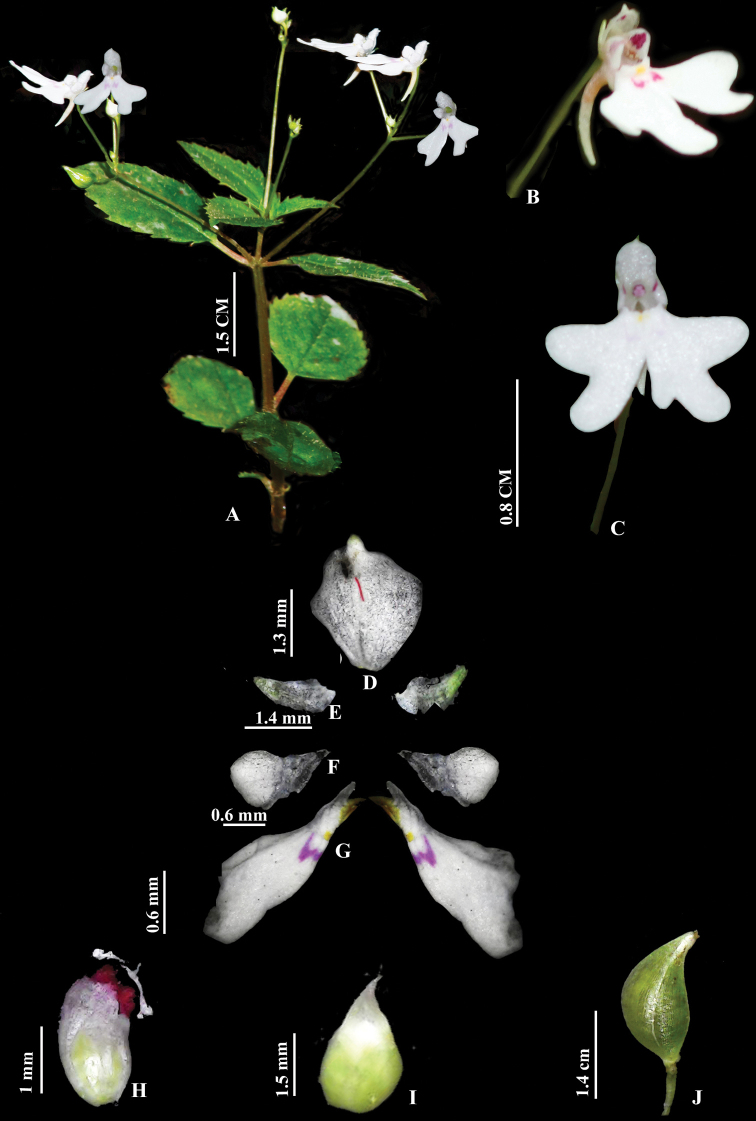
*Impatiensdanii***A** habit **B** flower (lateral view) **C** flower (front view) **D** dorsal petal **E** lateral sepal **F, G** lateral united petals **H** stamen **I** gynoecium **J** fruit. Photos by Govind.

#### Description.

Annual, erect herb, 10–20 cm high; stem simple to moderately branched, glabrous, often slightly pubescent terete, with few scattered, brown, sessile or stipitate glands, particularly in the lower part of the stem. Lower and middle leaves opposite, petiolate to subsessile; petiole up to 1.5 cm long; lamina ovate to ovoid or elliptic-lanceolate, 3.5–8.5 × 1–1.5 cm, base rounded with auricled lobes, apex acuminate, margins crenate, dentate to serrate or serrulate (usually in upper leaves) with cuspidate teeth; surfaces glabrous; upper leaves alternate, sessile, oblong-lanceolate, smaller than lower leaves, apex acuminate, surfaces glabrous or sometimes with few glands, particularly on lower surface. Inflorescence peduncled, 8–12 flowered racemes arising from the axis of alternate leaves in the upper part of the stem; peduncle up to 5 cm long, glabrous, with small brown spots; bracteoles absent; pedicels slender, 1–1.5 × 0.6–0.9 cm, glabrous, with or without sparse brown spots. Flowers 1–1.2 × 0.6–0.8 cm, white; with yellow-purple blotch at throat. Lateral sepals 2, opposite, one on either side, ovate to lanceolate, 1.3–1.8 × 0.6–0.8 mm, base cordate, unequally parted, margins entire, apex acute, surfaces glabrous with conspicuous purple dots. Dorsal petal orbicular to oblong, 2–3 × 1.5–2 mm, apex slightly notched, margins entire or wavy, concave in the middle with spreading sides, slightly keeled on dorsal side, 0.5–0.8 cm long. Lateral petals 2 lobed, lobes unequal with second lobe long and ovate, each lateral petal equal ca. 1–1.2 × 1.0–1.2 cm, margins (outer and inner) entire or wavy. Lower sepal saccate, white with curved spur. Spur equals the length of the lateral petal. Stamens 2–2.5 mm; filaments 1–1.5 mm long, anthers 0.8–1.5 mm long, partly fused; pollen grains bilateral 12 × 14 µm milky white. Ovary oblong-elliptic, 2–3 mm long, glabrous; style 0.1–0.4 mm long. Capsules ovoid, 1–2.8 cm long, 0.2–0.5 mm broad, glabrous, green with purplish base and apex, 4–10 seeded; seeds green, oblong or sub ovoid, 2–3.6 × 1.5–2 mm, surface covered with hairs.

**Figure 4.**
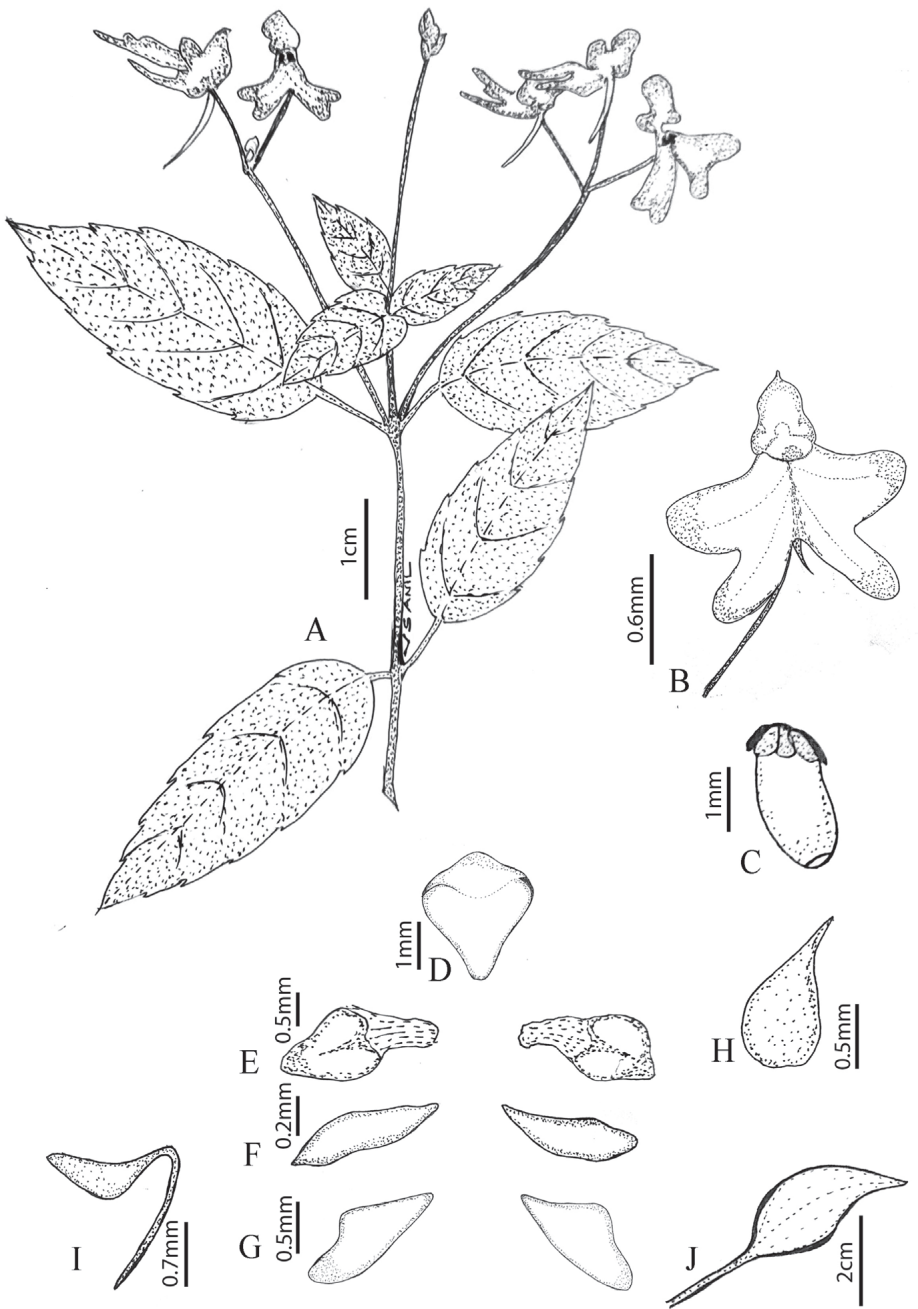
*Impatiensdanii***A** habit **B** flower **C** stamen **D** dorsal petal **E** basal lobe **F** lateral sepal **G** lateral united petals (distal lobe) **H** gynoecium **I** lower sepal with spur **J** fruit. Illustration by V.S. Anilkumar.

#### Etymology.

The specific epithet ‘*danii*’ is given in honor of Dr. Mathew Dan, Senior Scientist and Head, Plant Genetic Resource Division, Jawaharlal Nehru Tropical Botanic Garden and Research Institute, Thiruvananthapuram, Kerala, as a recognition of his immense contributions in the field of systematics and conservation of angiosperms in the Western Ghats.

#### Phenology.

August to November

#### Distribution and habitat.

*Impatiensdanii* is found to grow along the mud cliffs. Other taxa like *I.herbicola*, *I.munnarensis* and *Selaginellaciliaris* have been observed to grow along with this species.

#### Conservation status.

*Impatiensdanii* is known from a single location only (for a total of 50 individuals), and the AOO (Area of Occupancy) is 2 km^2^. On the basis of the IUCN Red List criteria (IUCN 2019) and the available data, we can apply the criteria B2 and C2ai and assess *I.danii* as Critically Endangered (CR).

#### Other notes.

*Impatiensdanii* belongs to the section Uniflorae, characterized by capsules that are short-fusiform, conspicuously turgid at middle, ca. 1 cm long, inflorescence a raceme with 2(–5) flowers and seed ellipsoid. Further the color of flower, blotches on throat, seed surface and shape of spur are distinct characters that delineate *I.danii* from other reported species.

#### Specimen examined.

*Impatiensdanii* India. Munnar, Idukki. 6 July 2020, M.G. Govind 987 (TBGT!). *I.goughii* India. Munnar, Idukki. 6 July 2020, M.G. Govind 988 (MH!).

### Impatiens shailajae

Taxon classificationPlantaeEricalesBalsaminaceae

Sindhu Arya & Kumar V.S.A. sp. nov.

51E8E37F-32CB-5617-868A-A581B9B96042

urn:lsid:ipni.org:names:77219075-1

Figs 5 , 6 , 7

#### Type.

India. Kerala, Thiruvananthapuram, Sangili, along the cliffs associated with streams of evergreen forest, 10.0889°N, 77.0595°E, 800 m a.s.l., 20 October 2020, Sindhu Arya & Kumar V.S.A.., 1088 (holotype TBGT!, isotype MH! CAL!).

#### Diagnosis.

*Impatiensshailajae* is similar to *I.minae* Ratheesh, Anil Kumar & Sivad. but differs with respect to the leaves (broadly ovate thin, rounded apex and green in *I.shailajae*vs. broadly ovate-orbicular, thick, fleshy, deep pink in *I.minae*), spur of the flower (straight and white in *I.shailajae* vs. slightly curved and pink in *I.minae*), lateral united petals (with white transparent papillae and small dorsal appendages in *I.shailajae* vs. red tipped white papillae and absence of dorsal appendages in *I.minae*) and seed (green with long bands of spiral hairs in *I.shailajae*vs. brown with short hairs in *I.minae*)

**Figure 5.**
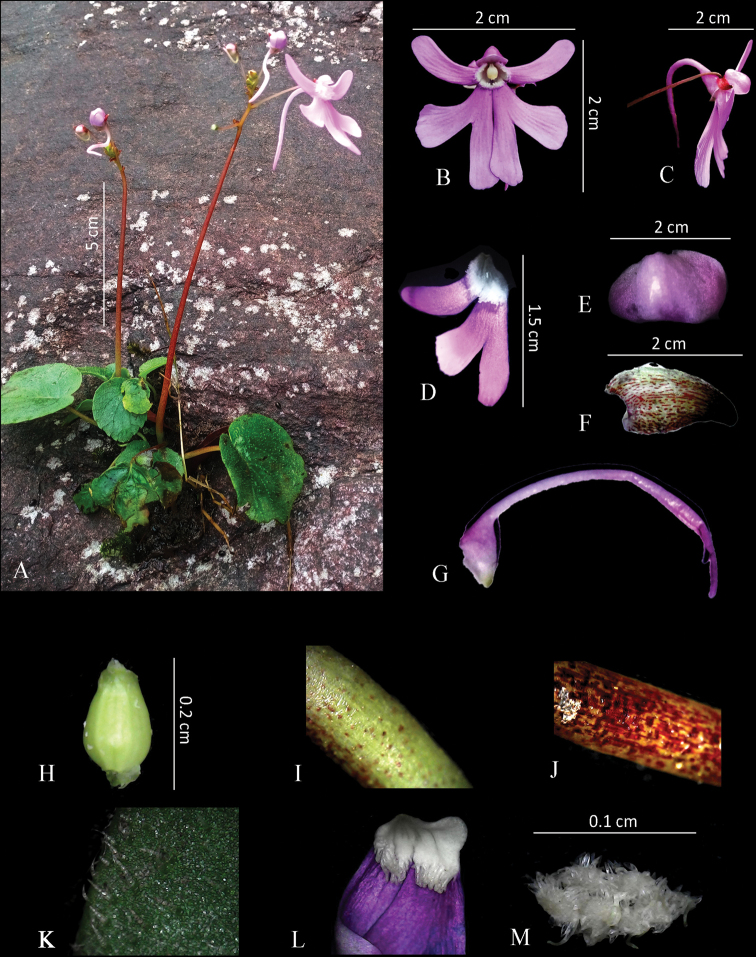
*Impatiensshailajae***A** habit **B** flower (front view) **C** flower (lateral view) **D** lateral petal **E** dorsal petal **F** lateral sepal **G** lower sepal with spur **H** gynoecium **I** scape lower surface **J** scape upper surface **K** leaf trichomes **L** papillae on petals **M** seed. Photos by Arya Sindhu.

#### Description.

Scapigerous terrestrial herbs, 10–15 cm high; rootstock faintly tuberous, lithophytic herbs, densely pubescent. Tubers oblate, 4–6 × 2–3 mm. Leaves 2–4, radical, fleshy, 4.5–5.5 × 6.5–7.2 cm, ovate-orbicular or reniform, obtuse or rounded at apex, base cordate, margin crenate or serrate, thickly hairy, dark green above, with a tuft of uniseriate trichomes on upper surface and silky lanuginose hairs on lower surface, nerves pale green, primary veins usually 8, palmate; petioles up to 2.5 cm long, light pink. Scape racemose, straight, 3–4 flowered 8–10 cm long, glabrous. Flowers clustered at the apex, violet, each c. 1.5 cm across; pedicels 1.0–1.5 cm long; bracts thick, broadly ovate, obovoid, 4–5.5 × 2.5–3.2 mm, yellowish with dark purple spots. Lateral sepals 2, each 3.0–4.0 × 2.0–2.2 mm; lower sepals long-spurred, spur slender, 1.0–1.5 cm long, milky white, straight. Dorsal petal broadly orbicular to obovoid, saccate, 5–6 × 5–7 mm, adaxially keeled, glabrous with pubescent keeled part, dull white to yellow or pale purple; keel mucronate, mucro ca. 1 mm long, pale green; lateral united petals 3–lobed, violet, with a slightly curved band of dense white tipped clavate papillae just above base; basal lobes shorter than the distal lobes, ca. 0.5 cm long, broadly oblong, rounded; middle lobes oblong towards tip, 4 mm long, broadly obovate; distal lobes spherical, ca. 6 mm long. Stamens 5, connate, 1.5 × 1.6 mm; filaments white, anthers white. Pollen grains 15 × 19 µm, light pink. Ovary green, 1.7–1.8 × 1.0–1.3 mm, elliptic, broadly acute at apex, glabrous. Capsule glabrous, reddish green, broadly ellipsoid, apex acute, 1.3–1.8 cm long. Seeds 5–8, ca. 1 mm long, surface with tuft of hairs.

**Figure 6.**
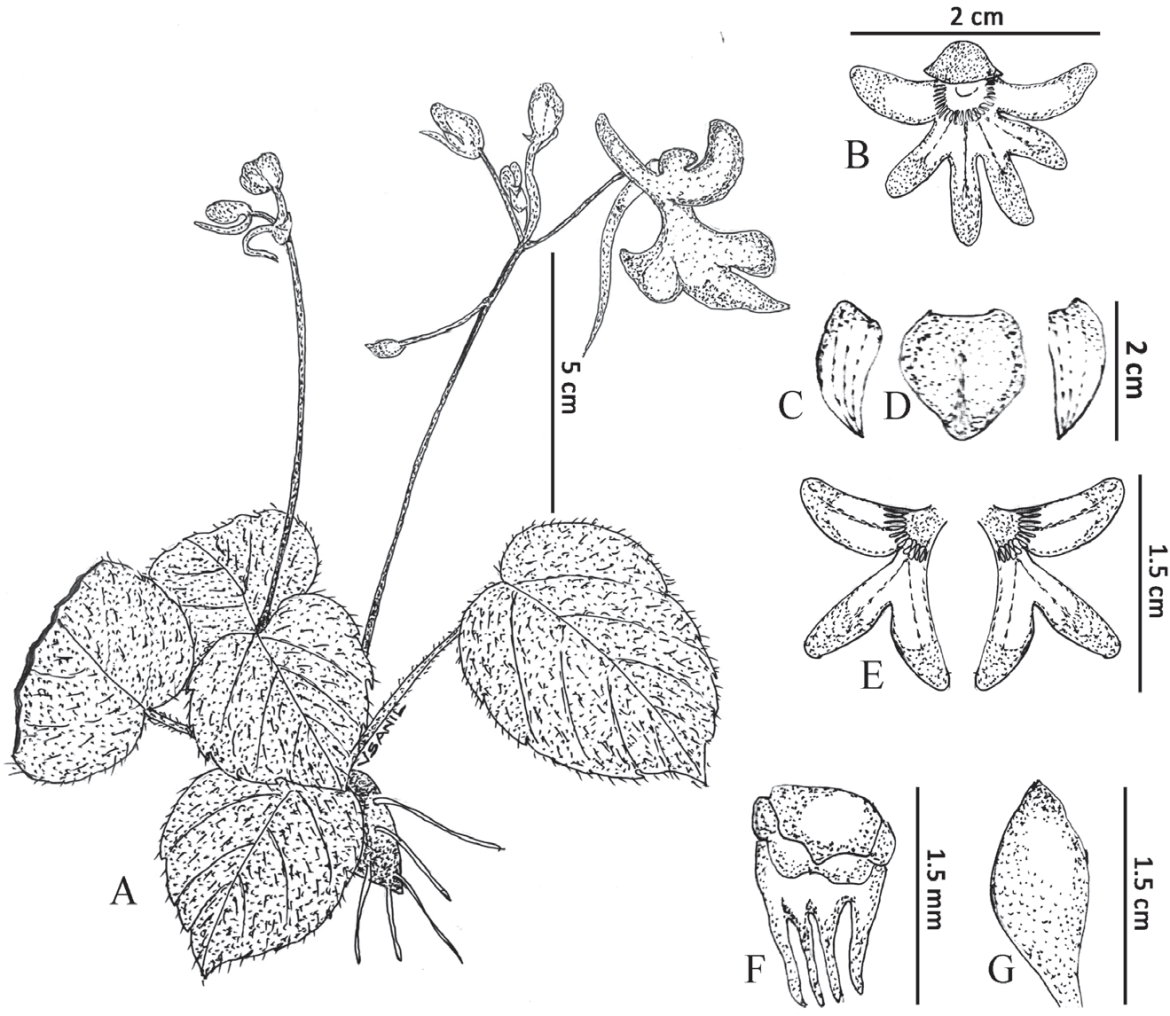
*Impatiensshailajae***A** habit **B** flower **C** lateral sepal **D** dorsal petal **E** lateral petal **F** stamen **G** gynoecium. Illustration by V.S. Anilkumar.

#### Etymology.

*Impatiensshailajae* is eponymous to Mrs. K.K. Shailaja, former Health Minister of Kerala, honoring her efforts to tackle various epidemic and pandemic situations in the state of Kerala through scientific temper.

#### Phenology.

August to November.

#### Distribution and habitat.

The species grows in the unexplored core forest area along steep slippery cliffs, continuously wet by water flow. The species is found to grow along with *I.verticillata* and *Fimbristylis* spp. in the near vicinity.

#### Conservation status.

The species was scattered in 3–4 population with 7–10 individuals per population. The population is well conserved without any disturbance as it was obtained from the interiors of protected forest. However, considering the lesser number of populations distributed across hardly 0.5 km, the species is assessed here as Critically Endangered (CE) by applying the criterion d (IUCN 2019).

#### Other notes.

*Impatiensshailajae* belongs to section Tuberosae, characterized by many-flowered racemose inflorescence; capsule clavate or linear, seed ellipsoid or ovoid and lateral sepals 4 with inner 2 fully developed (Yu et al. 2015). *Impatiensshailajae* also shares similarity with *I.scapiflora* but is distinct with respect to the color and shape of papillae on the standard petal, tuberous stolon and trichomes on the leaf. Further, the shape of dorsal petal and its keel is distinct in this new taxon which is easily visible at first glance. All these character combinations along with its undisturbed habitat (localized distribution) and micromorphology (prominent seed hair banding pattern and pollen morphology) further support the status of newly described species.

#### Specimen examined.

*Impatiensshailajae* India. Thiruvananthapuram Sangili, 15 October 2020, Arya & Kumar V.S.A. 2011 (MH!, TBGT!). *I.scapiflora* India. Idukki, 18 August 2019 Arya & Kumar V.S.A. 490 (TBGT!).

**Figure 7.**
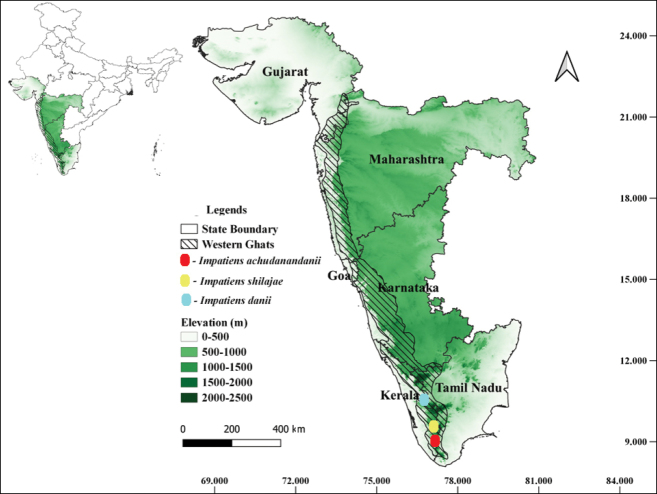
Distribution map of *Impatiensachudanandanii, Impatiensdanii* and *Impatiensshailajae*.

## Supplementary Material

XML Treatment for Impatiens achudanandanii

XML Treatment for Impatiens danii

XML Treatment for Impatiens shailajae

## References

[B1] Bhaskar V (2012) Taxonomic Monograph on *Impatiens* L. (Balsaminaceae) of Western Ghats, South India: the Key Genus for Endemism. Centre for plant taxonomic studies, Bangalore, 283 pp.

[B2] Bhaskar V Sringeswara AN (2017) Two new species of *Impatiens* L. under the section: ‘Annuae’ (Balsaminaceae) from Western Ghats, India. Webbia 72(2): 165–170. 10.1080/00837792.2017.1370208

[B3] Chhabra T Singh R Prabhukumar KM Hareesh VS (2016) Three new taxa of *Impatiens* (Balsaminaceae) from southern Western Ghats, India. Nordic Journal of Botany 34(6): 708–717. 10.1111/njb.01139

[B4] Dessai JRN Janarthanam MK (2011) The genus *Impatiens* (Balsaminaceae) in the northern and parts of central Western Ghats. Rheedea 21: 23–80.

[B5] Fischer E Rahelivololona ME (2015a) New taxa of *Impatiens* (Balsaminaceae) from Madagascar VI. *Impatiensotto-eleonorae*, a new species from Masoala Peninsula, and notes on the taxonomic relationships of *Impatiens firmula and I.hildebrandtii*. Phytotaxa 217(2): 155–163. 10.11646/phytotaxa.217.2.5

[B6] Fischer E Rahelivololona ME (2015b) New taxa of *Impatiens* (Balsaminaceae) from Madagascar IX. *Impatienslutzii*, a new species from Montagne d’Ambre National Park. Phytotaxa 239(2): 183–189. 10.11646/phytotaxa.239.2.7

[B7] Fischer E Rahelivololona ME (2015c) New taxa of *Impatiens* (Balsaminaceae) from Madagascar VII. Two new species of *Impatiens* from Mt. Marojejy, Madagascar. Phytotaxa 239(3): 213–222. 10.11646/phytotaxa.239.3.2

[B8] Fischer E Rahelivololona ME (2016) New taxa of *Impatiens* (Balsaminaceae) from Madagascar VIII. *Impatiensmax-huberi*, a new species from Marojejy and Anjanaharibe-Sud. Phytotaxa 244(2): e191. 10.11646/phytotaxa.244.2.7

[B9] Fischer E Rahelivololona ME Abrahamczyk S (2017) *Impatiensgalactica* (Balsaminaceae), a new spurless species of section Trimorphopetalum from Madagascar. Phytotaxa 298(3): e269. 10.11646/phytotaxa.298.3.6

[B10] Hareesh VS Sreekumar VB Dantas KJ Sujanapal P (2015) *Impatienssahyadrica* (Balsaminaceae), a new species from southern Western Ghats. Phytotaxa 207(3): 291–296. 10.11646/phytotaxa.207.3.8

[B11] Hooker JD (1875) *Impatiens*. In: Hooker JD (Ed.) Flora of British India. L.Reeve & Co, London 1: 440–464. 10.5962/bhl.title.54393

[B12] Hooker JD (1904–1906) An epitome of the British Indian species of *Impatiens*. Records of the Botanical Survey of India 4: 1–58.

[B13] Hooker JD (1908a) Les espèces du genre *Impatiens*. Nouvelles Archives du Museum d’Histoire Naturelle. Masson et Cie, Paris (France), ser. 4, 10: e246.

[B14] Hooker JD (1908b) *Impatiens*. Hooker’s Icones Plantarum. Dulau & Co, London, ser. 4, 9: 2851–2875.

[B15] Hooker JD (1910) Indian Species of *Impatiens*. Generis *Impatiens* Species Indicae Novae et Minus Rite Cognitae a Cl. A. Meebold detectae. Kew Bulletin Miscellaneous Information, Kew, 291–300. 10.2307/4111723

[B16] Hooker JD (1911) *Impatiens*. Hooker’s Icones Plantarum, ser. 4, 30: 2951–2975.

[B17] Hooker JD Thomson T (1860) Precursores ad Floram Indicum-Balsaminaceae. Journal Proceedings of Linnean Society of Botany 4(15): 106–157. 10.1111/j.1095-8339.1859.tb01160.x

[B18] IUCN (2019) Guidelines for using the IUCN Red List Categories and Criteria. Version 11. Prepared by the standards and petitions subcommittee. http://www.iucnredlist.org/documents/RedListGuidelines.pdf [accessed 12 January 2020]

[B19] Mabberley DJ (2008) Mabberley’s Plant-Book: A Portable Dictionary of Plants, Their Classification and Uses Utilizing Kubitzki’s The Families and Genera of Vascular Plants (1990–onwards) and Current Botanical Literature; Arranged According to The Principles of Molecular Systematics. Third Edition. Cambridge University Press, Cambridge.

[B20] Mani B Thomas S Britto SJ (2018) Two new species of *Impatiens* (Balsaminaceae) from the Western Ghats, India. Phytotaxa 334(3): 233–240. 10.11646/phytotaxa.334.3.4

[B21] Ramasubbu R Manikandan G Mehalingam P Pandurangan AG (2015) *Impatienscourtallensis* (Balsaminaceae), a new species of *Impatiens* from the Western Ghats, Tamil Nadu, India. Phytotaxa 203(2): 199–204. 10.11646/phytotaxa.203.2.10

[B22] Ramasubbu R Divya C Sasikala N Surendran A Sreekala AK (2017) *Impatiensmegamalayana*, a new species of *Impatiens* from the Western Ghats, Tamil Nadu, India. Phytotaxa 302(2): 193–197. 10.11646/phytotaxa.302.2.10

[B23] Song Y Yuan YM Kupfer P (2003) Chromosomal evolution in Balsaminaceae, with cytological observations on 45 species from Southeast Asia. Caryologia 56(4): 463–481. 10.1080/00087114.2003.10589359

[B24] Stevens PF (2012) Angiosperm Phylogeny Website, version 12. http://www.mobot.org/MOBOT/research/APweb/ [accessed 10 November 2016]

[B25] Thiers B (2021) [continuously updated] Index Herbariorum: A global directory of public herbaria and associated staff. New York Botanical Garden’s Virtual Herbarium. http://sweetgum.nybg.org/ih/ [accessed: 30 November 2019]

[B26] Vishnu M Venugopal DK Francis D Nampy S (2020) Two new scapigerous species of *Impatiens* (Balsaminaceae) from southern Western Ghats, India. Taiwania 2: e65.

[B27] Yu SX Janssens S Zhu XY Liden M Gao TG Wang W (2015) Phylogeny of Impatiens (Balsaminaceae): Integrating molecular and morphological evidence into a new classification. Cladistics 32(2): 1–19. 10.1111/cla.12119 34732016

[B28] Yuan YM Song Y Geuten K Rahelivololona E Wohlhauser S Fischer E Smets E Küpfer P (2004) Phylogeny and biogeography of Balsaminaceae inferred from ITS sequence data. Taxon 53(2): 391–403. 10.2307/4135617

